# 3-(4-Chloro­phenyl­sulfon­yl)-2,5-dimethyl-1-benzofuran

**DOI:** 10.1107/S1600536810044223

**Published:** 2010-11-06

**Authors:** Hong Dae Choi, Pil Ja Seo, Byeng Wha Son, Uk Lee

**Affiliations:** a Department of Chemistry, Dongeui University, San 24 Kaya-dong Busanjin-gu, Busan 614-714, Republic of Korea; b Department of Chemistry, Pukyong National University, 599-1 Daeyeon 3-dong, Nam-gu, Busan 608-737, Republic of Korea

## Abstract

In the title compound, C_16_H_13_ClO_3_S, the 4-chloro­phenyl ring makes a dihedral angle of 79.96 (5)° with the mean plane of the benzofuran fragment. In the crystal, mol­ecules are linked through weak inter­molecular C—H⋯O and C—H⋯π inter­actions.

## Related literature

For the biological activity of benzofuran compounds, see: Aslam *et al.* (2006 ▶); Galal *et al.* (2009 ▶); Khan *et al.* (2005 ▶). For natural products with benzofuran rings, see: Akgul & Anil (2003 ▶); Soekamto *et al.* (2003 ▶). For our previous structural studies of related 3-(4-chloro­phenyl­sulfin­yl)-2,5-dimethyl-1-benzofuran derivatives, see: Choi *et al.* (2010*a* ▶,*b* ▶).
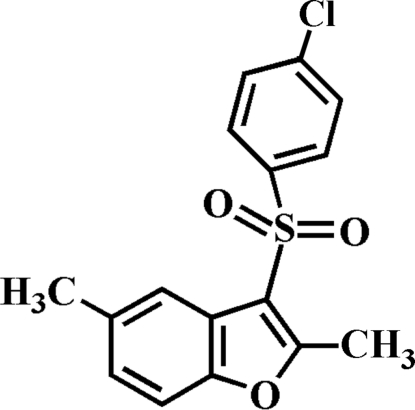


## Experimental

### 

#### Crystal data

C_16_H_13_ClO_3_S
*M* *_r_* = 320.77
Monoclinic, 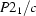

*a* = 15.3529 (3) Å
*b* = 12.1495 (2) Å
*c* = 8.2663 (2) Å
β = 105.363 (1)°
*V* = 1486.82 (5) Å^3^
*Z* = 4
Mo *K*α radiation
μ = 0.40 mm^−1^
*T* = 177 K
0.37 × 0.23 × 0.10 mm

#### Data collection

Bruker SMART APEXII CCD diffractometer
Absorption correction: multi-scan (*SADABS*; Bruker, 2009 ▶) *T*_min_ = 0.867, *T*_max_ = 0.962
13864 measured reflections
3398 independent reflections
2655 reflections with *I* > 2σ(*I*)
*R*_int_ = 0.033

#### Refinement

*R*[*F* ^2^ > 2σ(*F* ^2^)] = 0.040
*wR*(*F* ^2^) = 0.107
*S* = 1.06
3398 reflections
192 parameters
H-atom parameters constrained
Δρ_max_ = 0.29 e Å^−3^
Δρ_min_ = −0.34 e Å^−3^

### 

Data collection: *APEX2* (Bruker, 2009 ▶); cell refinement: *SAINT* (Bruker, 2009 ▶); data reduction: *SAINT*; program(s) used to solve structure: *SHELXS97* (Sheldrick, 2008 ▶); program(s) used to refine structure: *SHELXL97* (Sheldrick, 2008 ▶); molecular graphics: *ORTEP-3* (Farrugia, 1997 ▶) and *DIAMOND* (Brandenburg, 1998 ▶); software used to prepare material for publication: *SHELXL97*.

## Supplementary Material

Crystal structure: contains datablocks global, I. DOI: 10.1107/S1600536810044223/ng5054sup1.cif

Structure factors: contains datablocks I. DOI: 10.1107/S1600536810044223/ng5054Isup2.hkl

Additional supplementary materials:  crystallographic information; 3D view; checkCIF report

## Figures and Tables

**Table 1:** Hydrogen-bond geometry (Å, °) *Cg* is the centroid of the C2–C7 benzene ring.

*D*—H⋯*A*	*D*—H	H⋯*A*	*D*⋯*A*	*D*—H⋯*A*
C6—H6⋯O1^i^	0.95	2.56	3.495 (2)	167
C10—H10*A*⋯O2^ii^	0.98	2.43	3.279 (2)	145
C13—H13⋯*Cg*^iii^	0.95	2.74	3.411 (2)	128

Symmetry codes: (i) 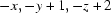; (ii) 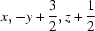; (iii) 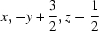.

**Fig. 1.**
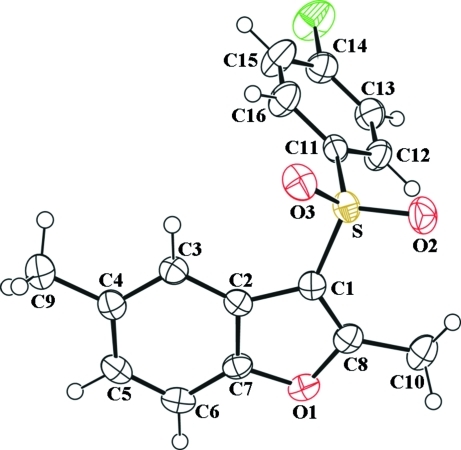
The molecular structure of the title compound with the atom numbering scheme. Displacement ellipsoids are drawn at the 50% probability level. H atoms are presented as a small spheres of arbitrary radius.

**Fig. 2.**
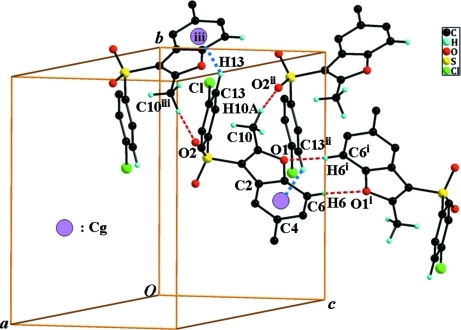
A view of the C–H···O and C–H···π interactions (dotted lines) in the crystal structure of the title compound. Cg denotes the centroid of the C2-C7 benzene ring. [Symmetry codes: (i) - x, - y + 1, - z + 2; (ii) x, - y +3/2, z + 1/2; (iii) x, - y + 3/2, z - 1/2.]

## supplementary crystallographic information

### Comment

A series of benzofuran ring system has received much attention in view of their particular pharmacological properties such as antifungal, antimicrobial, antitumor and antiviral activities (Aslam *et al.*, 2006; Galal *et al.*, 2009; Khan *et al.*, 2005). These compounds widely occur in nature (Akgul & Anil, 2003; Soekamto *et al.*, 2003). As a part of our study of the substituent effect on the solid state structures of 3-(4-chlorophenylsulfinyl)-2,5-dimethyl-1-benzofuran analogues (Choi *et al.*, 2010**a*,b*), we report herein on the crystal structure of the title compound.

In the title molecule (Fig. 1), the benzofuran unit is essentially planar, with a mean deviation of 0.003 (1 ) Å from the least-squares plane defined by the nine constituent atoms. The 4-chlorophenyl ring makes a dihedral angle of 79.96 (5)° with the mean plane of the benzofuran ring. The crystal packing (Fig. 2) is stabilized by weak intermolecular C–H···O hydrogen bonds: the first one between a benzene H atom and the furan O atom (Table 1, C6–H6···O1^i^), and the second one between a methyl H atom and the oxygen of the O═S═O unit (Table 1, C10–H10A···O2^ii^). The crystal packing (Fig. 2) is further stabilized by an intermolecular C–H···π interaction  between the 4-chlorophenyl H atom and the benzene ring (Table 1; C13–H13···Cg^iii^, Cg is the centroid of the C2–C7 benzene ring).

### Experimental

77% 3-chloroperoxybenzoic acid (269 mg, 1.2 mmol) was added in small portions to a stirred solution of 3-(4-chlorophenylsulfanyl)-2,5-dimethyl-1-benzofuran (346 mg, 1.2 mmol) in dichloromethane (40 mL) at 273 K. After being stirred at room temperature for 6h, the mixture was washed with saturated sodium bicarbonate solution and the organic layer was separated, dried over magnesium sulfate, filtered and concentrated at reduced pressure. The residue was purified by column chromatography (benzene) to afford the title compound as a colorless solid [yield 81%, m.p. 428-429 K; *R*_f_ = 0.59 (benzene)]. Single crystals suitable for X-ray diffraction were prepared by slow evaporation of a solution of the title compound in acetone at room temperature.

### Refinement

All H atoms were positioned geometrically and refined using a riding model, with C–H = 0.95 Å for aryl and  0.98 Å for methyl H atoms. *U*_iso_(H) = 1.2*U*_eq_(C) for aryl and 1.5*U*_eq_(C) for methyl H atoms.

### Crystal data

**Table:**

C_16_H_13_ClO_3_S	*F*(000) = 664
*M**_r_* = 320.77	*D*_x_ = 1.433 Mg m^−^^3^
Monoclinic, *P*2_1_/*c*	Mo *K*α radiation, λ = 0.71073 Å
Hall symbol: -P_2ybc	Cell parameters from 4847 reflections
*a* = 15.3529 (3) Å	θ = 2.8–27.5°
*b* = 12.1495 (2) Å	µ = 0.40 mm^−^^1^
*c* = 8.2663 (2) Å	*T* = 177 K
β = 105.363 (1)°	Block, colourless
*V* = 1486.82 (5) Å^3^	0.37 × 0.23 × 0.10 mm
*Z* = 4	

### Data collection

**Table:**

Bruker SMART APEXII CCD diffractometer	3398 independent reflections
Radiation source: rotating anode	2655 reflections with *I* > 2σ(*I*)
graphite multilayer	*R*_int_ = 0.033
Detector resolution: 10.0 pixels mm^-1^	θ_max_ = 27.5°, θ_min_ = 1.4°
φ and ω scans	*h* = −19→19
Absorption correction: multi-scan (*SADABS*; Bruker, 2009)	*k* = −15→14
*T*_min_ = 0.867, *T*_max_ = 0.962	*l* = −10→10
13864 measured reflections	

### Refinement

**Table:**

Refinement on *F*^2^	Primary atom site location: structure-invariant direct methods
Least-squares matrix: full	Secondary atom site location: difference Fourier map
*R*[*F*^2^ > 2σ(*F*^2^)] = 0.040	Hydrogen site location: difference Fourier map
*wR*(*F*^2^) = 0.107	H-atom parameters constrained
*S* = 1.06	*w* = 1/[σ^2^(*F*_o_^2^) + (0.0506*P*)^2^ + 0.4243*P*] where *P* = (*F*_o_^2^ + 2*F*_c_^2^)/3
3398 reflections	(Δ/σ)_max_ < 0.001
192 parameters	Δρ_max_ = 0.29 e Å^−^^3^
0 restraints	Δρ_min_ = −0.34 e Å^−^^3^

### Special details

**Table:**

Geometry. All esds (except the esd in the dihedral angle between two l.s. planes) are estimated using the full covariance matrix. The cell esds are taken into account individually in the estimation of esds in distances, angles and torsion angles; correlations between esds in cell parameters are only used when they are defined by crystal symmetry. An approximate (isotropic) treatment of cell esds is used for estimating esds involving l.s. planes.
Refinement. Refinement of F^2^ against ALL reflections. The weighted R-factor wR and goodness of fit S are based on F^2^, conventional R-factors R are based on F, with F set to zero for negative F^2^. The threshold expression of F^2^ > 2sigma(F^2^) is used only for calculating R-factors(gt) etc. and is not relevant to the choice of reflections for refinement. R-factors based on F^2^ are statistically about twice as large as those based on F, and R- factors based on ALL data will be even larger.

### Fractional atomic coordinates and isotropic or equivalent isotropic displacement parameters (Å^2^)

**Table:**

	*x*	*y*	*z*	*U*_iso_*/*U*_eq_	
Cl	0.48342 (4)	0.92660 (5)	0.73305 (10)	0.0705 (2)	
S	0.17974 (3)	0.58289 (4)	0.44925 (5)	0.03028 (14)	
O1	0.04560 (8)	0.56425 (10)	0.79164 (16)	0.0336 (3)	
O2	0.11294 (8)	0.63535 (12)	0.31700 (15)	0.0417 (3)	
O3	0.22166 (9)	0.48321 (11)	0.41515 (15)	0.0384 (3)	
C1	0.13384 (11)	0.55688 (14)	0.6156 (2)	0.0282 (4)	
C2	0.17212 (10)	0.48293 (14)	0.75339 (19)	0.0265 (4)	
C3	0.24701 (11)	0.41325 (14)	0.7986 (2)	0.0286 (4)	
H3	0.2872	0.4070	0.7293	0.034*	
C4	0.26171 (11)	0.35337 (15)	0.9461 (2)	0.0305 (4)	
C5	0.20127 (12)	0.36427 (16)	1.0465 (2)	0.0336 (4)	
H5	0.2120	0.3226	1.1471	0.040*	
C6	0.12720 (12)	0.43281 (15)	1.0054 (2)	0.0346 (4)	
H6	0.0871	0.4400	1.0748	0.041*	
C7	0.11476 (11)	0.49046 (15)	0.8570 (2)	0.0287 (4)	
C8	0.05880 (11)	0.60332 (15)	0.6451 (2)	0.0312 (4)	
C9	0.34169 (13)	0.27807 (18)	0.9993 (2)	0.0426 (5)	
H9A	0.3251	0.2045	0.9527	0.064*	
H9B	0.3608	0.2738	1.1220	0.064*	
H9C	0.3914	0.3068	0.9579	0.064*	
C10	−0.00891 (12)	0.68520 (16)	0.5565 (3)	0.0403 (5)	
H10A	0.0030	0.7562	0.6145	0.060*	
H10B	−0.0696	0.6598	0.5558	0.060*	
H10C	−0.0048	0.6936	0.4409	0.060*	
C11	0.26643 (11)	0.67929 (14)	0.5303 (2)	0.0285 (4)	
C12	0.24350 (12)	0.78759 (16)	0.5522 (2)	0.0372 (4)	
H12	0.1818	0.8092	0.5243	0.045*	
C13	0.31025 (13)	0.86365 (16)	0.6145 (3)	0.0409 (5)	
H13	0.2954	0.9381	0.6301	0.049*	
C14	0.39885 (13)	0.83017 (17)	0.6538 (3)	0.0418 (5)	
C15	0.42284 (13)	0.72285 (18)	0.6332 (3)	0.0527 (6)	
H15	0.4846	0.7016	0.6616	0.063*	
C16	0.35555 (12)	0.64652 (17)	0.5705 (3)	0.0435 (5)	
H16	0.3706	0.5721	0.5552	0.052*	

### Atomic displacement parameters (Å^2^)

**Table:**

	*U*^11^	*U*^22^	*U*^33^	*U*^12^	*U*^13^	*U*^23^
Cl	0.0463 (3)	0.0523 (4)	0.1062 (6)	−0.0155 (3)	0.0085 (3)	−0.0142 (3)
S	0.0351 (2)	0.0300 (3)	0.0243 (2)	0.00103 (18)	0.00535 (17)	0.00088 (17)
O1	0.0275 (6)	0.0353 (7)	0.0396 (7)	−0.0011 (5)	0.0114 (5)	−0.0041 (6)
O2	0.0411 (7)	0.0475 (9)	0.0302 (7)	0.0000 (6)	−0.0013 (5)	0.0064 (6)
O3	0.0524 (8)	0.0333 (7)	0.0313 (6)	0.0023 (6)	0.0143 (6)	−0.0041 (6)
C1	0.0294 (8)	0.0253 (9)	0.0285 (8)	−0.0018 (7)	0.0050 (7)	−0.0015 (7)
C2	0.0294 (8)	0.0247 (9)	0.0251 (8)	−0.0053 (7)	0.0066 (6)	−0.0032 (7)
C3	0.0312 (8)	0.0280 (9)	0.0281 (8)	0.0004 (7)	0.0105 (7)	−0.0024 (7)
C4	0.0328 (9)	0.0276 (9)	0.0294 (8)	−0.0032 (7)	0.0050 (7)	−0.0005 (7)
C5	0.0402 (10)	0.0331 (10)	0.0276 (8)	−0.0092 (8)	0.0090 (7)	0.0012 (8)
C6	0.0358 (9)	0.0377 (11)	0.0340 (9)	−0.0083 (8)	0.0160 (8)	−0.0043 (8)
C7	0.0254 (8)	0.0279 (9)	0.0333 (9)	−0.0036 (7)	0.0086 (7)	−0.0050 (7)
C8	0.0288 (8)	0.0283 (9)	0.0338 (9)	−0.0042 (7)	0.0039 (7)	−0.0053 (8)
C9	0.0444 (11)	0.0420 (12)	0.0403 (10)	0.0056 (9)	0.0092 (9)	0.0095 (9)
C10	0.0325 (9)	0.0334 (11)	0.0498 (11)	0.0038 (8)	0.0016 (8)	−0.0051 (9)
C11	0.0325 (8)	0.0277 (9)	0.0254 (8)	0.0027 (7)	0.0076 (7)	0.0041 (7)
C12	0.0316 (9)	0.0315 (10)	0.0471 (11)	0.0065 (8)	0.0082 (8)	0.0035 (9)
C13	0.0429 (10)	0.0281 (10)	0.0522 (12)	0.0023 (8)	0.0134 (9)	0.0013 (9)
C14	0.0361 (10)	0.0380 (11)	0.0490 (11)	−0.0068 (8)	0.0074 (8)	−0.0018 (9)
C15	0.0299 (10)	0.0440 (13)	0.0790 (16)	0.0052 (9)	0.0055 (10)	−0.0068 (12)
C16	0.0358 (10)	0.0337 (11)	0.0591 (13)	0.0073 (8)	0.0092 (9)	−0.0028 (10)

### Geometric parameters (Å, °)

**Table:**

Cl—C14	1.743 (2)	C6—H6	0.9500
S—O3	1.4339 (14)	C8—C10	1.484 (2)
S—O2	1.4350 (13)	C9—H9A	0.9800
S—C1	1.7319 (17)	C9—H9B	0.9800
S—C11	1.7664 (18)	C9—H9C	0.9800
O1—C8	1.366 (2)	C10—H10A	0.9800
O1—C7	1.385 (2)	C10—H10B	0.9800
C1—C8	1.362 (2)	C10—H10C	0.9800
C1—C2	1.448 (2)	C11—C16	1.378 (2)
C2—C7	1.384 (2)	C11—C12	1.386 (3)
C2—C3	1.397 (2)	C12—C13	1.375 (3)
C3—C4	1.386 (2)	C12—H12	0.9500
C3—H3	0.9500	C13—C14	1.374 (3)
C4—C5	1.406 (2)	C13—H13	0.9500
C4—C9	1.501 (2)	C14—C15	1.378 (3)
C5—C6	1.377 (3)	C15—C16	1.383 (3)
C5—H5	0.9500	C15—H15	0.9500
C6—C7	1.381 (3)	C16—H16	0.9500
			
O3—S—O2	119.53 (8)	C4—C9—H9A	109.5
O3—S—C1	107.47 (8)	C4—C9—H9B	109.5
O2—S—C1	109.07 (8)	H9A—C9—H9B	109.5
O3—S—C11	107.60 (8)	C4—C9—H9C	109.5
O2—S—C11	108.01 (8)	H9A—C9—H9C	109.5
C1—S—C11	104.12 (8)	H9B—C9—H9C	109.5
C8—O1—C7	107.00 (13)	C8—C10—H10A	109.5
C8—C1—C2	107.60 (15)	C8—C10—H10B	109.5
C8—C1—S	127.45 (14)	H10A—C10—H10B	109.5
C2—C1—S	124.91 (13)	C8—C10—H10C	109.5
C7—C2—C3	119.21 (15)	H10A—C10—H10C	109.5
C7—C2—C1	104.70 (15)	H10B—C10—H10C	109.5
C3—C2—C1	136.09 (15)	C16—C11—C12	120.86 (17)
C4—C3—C2	118.96 (15)	C16—C11—S	119.99 (14)
C4—C3—H3	120.5	C12—C11—S	119.15 (13)
C2—C3—H3	120.5	C13—C12—C11	119.80 (17)
C3—C4—C5	119.40 (16)	C13—C12—H12	120.1
C3—C4—C9	120.51 (16)	C11—C12—H12	120.1
C5—C4—C9	120.09 (16)	C14—C13—C12	118.87 (18)
C6—C5—C4	122.84 (16)	C14—C13—H13	120.6
C6—C5—H5	118.6	C12—C13—H13	120.6
C4—C5—H5	118.6	C13—C14—C15	122.10 (18)
C5—C6—C7	115.89 (16)	C13—C14—Cl	118.84 (16)
C5—C6—H6	122.1	C15—C14—Cl	119.06 (15)
C7—C6—H6	122.1	C14—C15—C16	118.91 (18)
C6—C7—C2	123.71 (16)	C14—C15—H15	120.5
C6—C7—O1	125.86 (15)	C16—C15—H15	120.5
C2—C7—O1	110.43 (15)	C11—C16—C15	119.46 (18)
C1—C8—O1	110.27 (15)	C11—C16—H16	120.3
C1—C8—C10	134.82 (17)	C15—C16—H16	120.3
O1—C8—C10	114.91 (15)		
			
O3—S—C1—C8	−148.80 (16)	C8—O1—C7—C2	0.37 (18)
O2—S—C1—C8	−17.84 (19)	C2—C1—C8—O1	−0.30 (19)
C11—S—C1—C8	97.26 (17)	S—C1—C8—O1	−178.16 (12)
O3—S—C1—C2	33.69 (16)	C2—C1—C8—C10	178.95 (18)
O2—S—C1—C2	164.64 (14)	S—C1—C8—C10	1.1 (3)
C11—S—C1—C2	−80.26 (15)	C7—O1—C8—C1	−0.03 (18)
C8—C1—C2—C7	0.51 (18)	C7—O1—C8—C10	−179.45 (14)
S—C1—C2—C7	178.44 (13)	O3—S—C11—C16	−8.62 (18)
C8—C1—C2—C3	−179.35 (18)	O2—S—C11—C16	−138.91 (15)
S—C1—C2—C3	−1.4 (3)	C1—S—C11—C16	105.24 (16)
C7—C2—C3—C4	0.1 (2)	O3—S—C11—C12	171.21 (14)
C1—C2—C3—C4	179.94 (18)	O2—S—C11—C12	40.92 (17)
C2—C3—C4—C5	−0.1 (2)	C1—S—C11—C12	−74.93 (16)
C2—C3—C4—C9	−179.70 (16)	C16—C11—C12—C13	0.1 (3)
C3—C4—C5—C6	−0.2 (3)	S—C11—C12—C13	−179.71 (14)
C9—C4—C5—C6	179.34 (17)	C11—C12—C13—C14	0.0 (3)
C4—C5—C6—C7	0.6 (3)	C12—C13—C14—C15	−0.2 (3)
C5—C6—C7—C2	−0.6 (3)	C12—C13—C14—Cl	−179.91 (15)
C5—C6—C7—O1	−179.53 (16)	C13—C14—C15—C16	0.2 (4)
C3—C2—C7—C6	0.3 (3)	Cl—C14—C15—C16	179.91 (18)
C1—C2—C7—C6	−179.60 (16)	C12—C11—C16—C15	−0.1 (3)
C3—C2—C7—O1	179.35 (14)	S—C11—C16—C15	179.72 (17)
C1—C2—C7—O1	−0.54 (18)	C14—C15—C16—C11	0.0 (3)
C8—O1—C7—C6	179.41 (17)		

### Hydrogen-bond geometry (Å, °)

**Table:**

Cg is the centroid of the C2–C7 benzene ring.

**Table:**

*D*—H···*A*	*D*—H	H···*A*	*D*···*A*	*D*—H···*A*
C6—H6···O1^i^	0.95	2.56	3.495 (2)	167
C10—H10A···O2^ii^	0.98	2.43	3.279 (2)	145
C13—H13···Cg^iii^	0.95	2.74	3.411 (2)	128

Symmetry codes: (i) −*x*, −*y*+1, −*z*+2; (ii) *x*, −*y*+3/2, *z*+1/2; (iii) *x*, −*y*+3/2, *z*−1/2.
